# How to Determine if a Project Is Human Subjects Research, a Quality Improvement Project, or Both

**DOI:** 10.31486/toj.19.0087

**Published:** 2020

**Authors:** Pat F. Bass, John W. Maloy

**Affiliations:** ^1^ Office of Research, Louisiana State University Health Sciences Center Shreveport, Shreveport, LA; ^2^ Assistant Vice Chancellor for Research Management, Louisiana State University Health Sciences Center Shreveport, Shreveport, LA

**Keywords:** *Quality improvement*, *quality of healthcare*, *research subjects*

## Abstract

**Background:** Before undertaking a research project, investigators must determine if the planned activity is human subjects research or a quality improvement project because specific regulations govern the conduct of human subjects research. Making this determination, however, can be confusing because human subjects research and quality improvement projects share similar characteristics.

**Methods:** The definitions, questions, and examples provided in this article will help investigators decide between quality improvement projects and human subjects research or determine when to seek regulatory guidance.

**Results:** While quality improvement and human subjects research are both rigorous processes and at times involve similar methods, the two types of studies have distinctly different overall aims. Quality improvement projects use data-driven methods to improve health delivery and quality. Such projects examine changes in human behavior and are largely experiential learning processes. Research is a systematic investigation designed to develop or contribute to generalizable knowledge.

**Conclusion:** In most instances, the goals of human subjects research and quality improvement projects do not intersect, and quality improvement projects are generally not subject to US Department of Health and Human Services (HHS) regulatory protections. However, some projects are both quality improvement and human subjects research, and sometimes, a quality improvement project develops into a human subjects research project. Investigators must be aware of the criteria defining human subjects research to ensure that HHS regulations for the protection of human subjects are applied when necessary.

## INTRODUCTION

Improving the quality of care is intrinsic to the daily activities of any good practitioner of medicine. Imagine the following primary care practice scenarios:

- You want to reduce vaccination errors. You decide to implement a no-interruption process during vaccination preparation and administration.
- You notice that diabetic patients on a certain combination treatment plan develop arthritis more commonly than patients receiving an alternative therapy. After finding little information about this scenario in the medical literature, you decide to test a specific exercise regimen to determine if it decreases arthritis symptoms in these patients.

In both instances, you want to share the results of your project locally and at a regional professional meeting. Are these activities quality improvement or human subjects research? The investigator must determine the appropriate classification for a project because specific protection regulations apply to human subjects research.^1^ Making this determination, however, can be confusing because human subjects research and quality improvement projects share similar characteristics.^2^ They both

- ask clinically important questions
- use patient and hospital data
- involve analysis of data collected as part of a project
- may involve direct interactions with patients
- seek to improve patient care or experience for patients and providers

While quality improvement and human subjects research are both rigorous processes and at times involve similar methods, quality improvement and human subjects research have distinctly different overall aims. Quality improvement projects use data-driven methods to improve health delivery and quality. These projects examine changes in human behavior and are largely experiential learning processes. Research is a systematic investigation designed to develop or contribute to generalizable knowledge.

The following questions can help an investigator determine if a particular activity is human subjects research and therefore subject to human subjects protection regulations^3^:

1. Does the activity involve research according to definitions outlined in the Code of Federal Regulations at 45 CFR §46.102(d)?
2. Are human subjects involved as defined in 45 CFR §46.102(f)?
3. Does the research qualify for an exemption under 45 CFR §46.101(b)?
4. Is the project nonexempt human subjects research supported by the US Department of Health and Human Services (HHS) or otherwise covered by an institution's Federalwide Assurance (the required federal documentation of an institution's commitment to comply with federal regulations and maintain policies and procedures for the protection of human participants)?

If an investigator can clearly answer yes to the preceding questions, the project is most likely subject to the human subjects research regulations of HHS. However, most quality improvement activities will not meet these criteria, and the information in this article will help the investigator decide between quality improvement projects and human subjects research or determine when to seek regulatory guidance.

## HUMAN SUBJECTS RESEARCH DEFINED

Whether a project involves human subjects should be the first question a researcher asks. A human subject is defined in 45 CFR §46.102 as a “living individual about whom an investigator, whether professional or student, conducting research:

- obtains information or biospecimens through intervention or interaction with the individual, and uses, studies, or analyzes the information or biospecimens; or
- obtains, uses, studies, analyzes, or generates identifiable private information or identifiable biospecimens.”^1^

Research is defined by federal regulations in 45 CFR §46.102 as “a systematic investigation, including research development, testing, and evaluation, designed to develop or contribute to generalizable knowledge.”^1^ Research projects involving human subjects require review by an institutional review board (IRB).

Medical research is generally designed to test a hypothesis and follows a formal protocol. A research project has at least one objective, and the investigator outlines a set of procedures that are strictly followed to achieve that objective. The researcher is often seeking to generalize findings to a specific population beyond the specific patients or institution where the research is performed. The researcher is bound by regulatory policies and must obtain informed consent from participants or a waiver of consent from the IRB.

## QUALITY IMPROVEMENT DEFINED

Quality improvement, also sometimes referred to as quality assurance, is also systematic, but the goal is to improve care, processes, or outcomes in an organization. A straightforward definition of quality improvement is “a systematic, data-guided activity designed to bring about immediate improvement in a local setting.”^4^ Quality improvement is not defined in the Federal Policy for the Protection of Human Subjects—the Common Rule—but the Centers for Medicare and Medicaid Services (CMS) defines quality improvement in 42 CFR §480.101 as “an assessment, conducted by or for a quality improvement organization, of a patient care problem for the purpose of improving patient care through peer analysis, intervention, resolution of the problem and follow-up.”^5^ CMS further elaborates in 42 CFR §476.1: “Quality improvement initiative means any formal activity designed to serve as a catalyst and support for quality improvement that uses proven methodologies to achieve these improvements. The improvements may relate to safety, health care, health and value and involve providers, practitioners, beneficiaries, and/or communities.”^5^

Quality improvement projects commonly involve the implementation and testing of new processes using the Plan-Do-Study-Act (PDSA) process. As the PDSA cycle is repeated, changes are made to continue to improve the outcome.

## DISTINGUISHING BETWEEN HUMAN SUBJECTS RESEARCH AND QUALITY IMPROVEMENT PROJECTS

While identifying a quality improvement project may be straightforward, determining if a project is both quality improvement and human subjects research is more challenging. The Table lists common descriptions and characteristics that may help with this determination.^4–7^

**Table.** Comparison of Human Subjects Research and Quality Improvement Projects^4–7^

Study Design Element	Human Subjects Research Project	Quality Improvement Project
Purpose	Gather facts to test a hypothesis and develop or contribute to generalizable knowledge.	Improve and understand specific, local processes or practices commonly related to cost, productivity, operations, quality, or patient experience.
Starting point	Answer a question or test a hypothesis that can be applied to a more general population.	Improve performance in a specific unit or population (patient or provider) in an organization.
Design	Systematic design with strict adherence to a protocol that does not change throughout the process. May involve randomization.	Iterative and adaptive design that may or may not be systematic. Usually does not involve randomization.
Beneficiaries	Clinician, researcher, scientific community, and occasionally the subject benefit. Results do not directly benefit institutional practice or programs.	Patients, staff, providers, and institution benefit.
Mandate	Institutions do not mandate research activities or programs.	Activities are usually mandated by institutions or clinics as part of clinical operations.
Impact	Designed to contribute to generalizable knowledge and may or may not benefit subjects.	Findings are expected to directly impact institutional processes or practices.
Measures	Measurement instruments must have estimates of reliability, validity, specificity, and sensitivity. Instruments are often complex and have a significant time burden. Protocols are followed closely, and confounding variables are measured or controlled for. Studies may occur over long periods of time (years).	Measurement instruments are generally limited, simple, easy to administer, and not overburdensome to the provision of care. Iterative, rapid cycles are followed, and confounding variables are acknowledged but not measured. Timeline is commonly weeks to months.
Adoption of results	Little urgency to disseminate results quickly.	Results rapidly adopted into local care delivery.
Participants	Subset of a population without an obligation to participate. Participants must meet strict inclusion and exclusion criteria. Investigator will calculate a sample size to determine how many participants are needed.	Most or all of the population involved in the process or practice. The responsibility to participate is a component of care, and the expectation is that most individuals participate.
Benefits	Participants may or may not benefit directly.	Direct benefit to system, program, or process is expected although participants may not receive direct benefit.
Risks	Subjects may be placed at risk, and risks are stated in the informed consent document.	By design, does not increase patients’ risk, with the exception of possible privacy/confidentiality concerns. Consent is implied as part of care.
Analysis	An a priori hypothesis is developed by the researcher to be statistically proved or disproved.	A program, process, or system is compared to an established set of standards, outcomes, or targets.
Outcome	Answer a research question and statistically prove or disprove a hypothesis. Significant scientific rigor is applied.	Promptly improve a program/process/system after comparison with an established set of standards. Process validity is sought.
Dissemination of results	Intent to disseminate assumed at the outset of the project with results expected to develop or contribute to generalizable knowledge by filling a gap in the scientific literature.	Intent to disseminate is not assumed at the outset of the project and often does not occur beyond the institution; when results are published, the intent is to suggest potentially effective models and strategies rather than generalizable knowledge.
Use of placebo	Use of placebo may be planned.	Comparison of standard treatments, practices, techniques, or processes. Placebo is not used.
Deviation from standard practice	May involve significant deviation from standard practice.	Unlikely to involve significant deviation from standard practice.

At a basic level, research is about the discovery of new information, while quality improvement primarily emphasizes the application of known knowledge.^7^ One of the principal distinguishing differences between quality improvement and human subjects research is exposure to risk. With the exception of loss of privacy and confidentiality, quality improvement projects should not expose patients to more than minimal risk. The assumption is that quality improvement activities are at least as safe as routine care.^2^ On the other hand, risk to human subjects is approved by the IRB and outlined in the informed consent document, giving potential research participants the opportunity to decide if they wish to enroll in the study or not. IRB review demonstrates to the medical community that the project is thoughtful and well designed, the risks are reasonable in relation to the anticipated benefits, and the knowledge expected to result from the study is important.^2^

Some research projects may qualify for an IRB exemption, some projects definitely require full IRB review, and some projects may not meet the criteria for human subjects research.

### Projects That May Qualify for Institutional Review Board Exemption

Only the IRB is allowed to decide if a project is exempt. If a project is exempt, certain federal regulations such as annual continuing review and IRB notification of study closure do not apply. Examples of exempt human subjects research are projects that use or focus on the following:

- Established educational practices
- Educational tests or survey procedures
- Existing data
- Public benefit or service programs evaluation
- Taste and food quality^8^

In the healthcare setting, examples of human subjects research projects that require IRB review but may qualify for an exemption include the following:

- Administering questionnaires or interviewing human subjects for the purposes of quantitative analysis or a mixed qualitative/quantitative analysis
- Using bodily materials—such as cells, blood, urine, or tissues even if these materials were not collected specifically for the study—that are coded so that the data is nonidentifiable and the investigator does not have access to the coding key
- Conducting medical record reviews

### Projects That Are Human Subjects Research

Projects that do not meet one of the exemption criteria are human subjects research and require IRB review. Examples include the following:

- Using human subjects to evaluate a new device, product, or drug
- Collecting data via intervention or interaction with an individual
- Collecting or using private information that can readily identify a participant
- Producing generalizable information that uses individually identifiable information^8^

### Projects That Are Not Human Subjects Research

Examples of studies that are not human subjects research and may not require IRB review include the following^8^:

- Data collection for internal administration purposes such as teaching evaluations or employee satisfaction surveys.
- Data collection for internal use to improve departmental programs or services. If the data are later determined to be generalizable, IRB review may be required before analysis and publication.
- General information gathering or program evaluation activities focused on policies and procedures, such as asking researchers about the impacts of a new institutional policy (eg, the impact on research programs of increasing the indirect cost rate).
- Oral history research that is not generalizable beyond an individual.
- Independent contracts for activities carried out by an external agency such as cost-benefit analyses or customer satisfaction studies.
- Research involving cadavers, autopsy material, or biospecimens from deceased individuals.
- Quality improvement projects unless the project is intended to contribute to generalizable knowledge.
- Case reports if the case is limited to a description of the clinical features and does not contribute to generalizable knowledge. The number of cases needed to meet the criteria as research is not defined in the Code of Federal Regulations; therefore, most IRBs locally define the number of cases that do not constitute human subjects research, as well as a threshold number when systematic investigation status is reached.
- Publicly available data such as census data or cancer statistics.

As noted previously, quality improvement projects are generally not considered human subjects research unless the intent to contribute to generalizable knowledge is clear. Examples of quality improvement projects and activities that are not likely to meet the definition of human subjects research include the following:

- Implementing a practice (eg, a no-interruption process as described in the Introduction) to improve some aspect of quality related to patient care
- Collecting provider or patient data for the purpose of evaluating program implementation, patient or provider satisfaction, or clinical effectiveness related to a practice
- Collecting administrative data related to a practice
- Measuring and reporting provider or staff performance for clinical or administrative purposes

## PROJECTS THAT ARE BOTH QUALITY IMPROVEMENT AND HUMAN SUBJECTS RESEARCH

Projects can sometimes both be quality improvement and human subjects research. Examples include the following^4,6^:

- Implementation of an untested clinical intervention with the dual purpose of not only improving the quality of care but also collecting information about generalizable patient outcomes. If data are collected to establish scientific evidence involving how well an intervention achieves intended results, a quality improvement project may also constitute nonexempt human subjects research that requires IRB review. If the project team anticipates this outcome ahead of project initiation, IRB approval should be sought prior to beginning the project.
- A collaborative multisite quality improvement project with the goal of improving some aspect of care. If the data are analyzed for generalizable knowledge, the activity is likely human subjects research.
- Evaluation of patient data to develop a new clinical treatment guideline.
- Development of new quality assessment or measurement techniques and tools.
- A project that randomizes participants into three different interventions to improve medication compliance. All three arms are designed to improve care, but the investigators do not know which arm is best.
- Funding, significant involvement/participation, or sponsorship by an entity outside of the organization where the activity occurs. While funding alone does not automatically classify a project as human subjects research, many institutions commonly receive funding expressly for research projects.
- Key personnel in the project have no ongoing commitment/involvement to the care being provided.
- Delaying feedback from monitoring changes so that data are not biased.

## PROJECTS THAT BEGIN AS QUALITY IMPROVEMENT BUT BECOME HUMAN SUBJECTS RESEARCH

When an investigator designs a quality improvement project with the idea that the activity is also human subjects research, the investigator should submit a protocol for IRB review before initiating the project. However, an investigator may realize after initiating or completing a quality improvement project that further study would make the results generalizable. Cases such as this generally require submission to the IRB for a secondary use of data. Examples include the following:

- A quality improvement project using biologic samples is implemented to determine which diagnostic test is best for a particular diagnosis at a local facility.
- A surgeon implements a certain technique in his/her practice and tracks the results. The surgeon decides the results may benefit others, so he/she wants to systematically analyze and generalize the outcomes. IRB review is required prior to review of the gathered data as the surgeon is now trying to generalize outcomes.
- A faculty member begins a new educational program to improve students’ academic achievement. After several classes in which the program was a success, the instructor decides to share the results. IRB review is required before the instructor can analyze the previously collected data.

## QUESTIONS TO HELP WITH THE DECISION

By applying the following questions, investigators can assess if a project is human subjects research, a quality improvement project, or both^4,6,9^:

1. Do I intend this project to lead to generalizable knowledge? A number of factors may come into play. If the intent of a project is program evaluation or practice improvement, or if the project is only designed to evaluate local and not broader outcomes, the project outcomes are likely not generalizable. However, the design or structure of a project may indicate a desire for generalizability. For example, using systematic research methodologies to ensure external validity and reproducibility of results makes a project more likely to be human subjects research than a quality improvement project. On the other hand, if the project goal is to simply report what happened at an institution following implementation of a process or procedure, such a design does not signal the intention to generalize results beyond the single institution. Similarly, a design that allows for continuous cycles of improvement, such as PDSA cycles, is indicative of a quality improvement project. The intent to publish does not classify a project as human subjects research.
2. Will the project lead to change in specific institutional or programmatic practices? If the primary aim of the project is to improve care or operational efficiencies, the project is more likely a quality improvement project rather than human subjects research.
3. Is the project intended to improve a process or delivery of care in a specific healthcare setting? If so, the project is more likely to be a quality improvement project rather than human subjects research.
4. What are the project methods? Control groups, randomization, and a fixed protocol are consistent with a research design. In contrast, flexible and customizable methods that incorporate rapid evaluation, feedback cycles, and incremental changes are consistent with quality improvement design. Additionally, interventions in a quality improvement project are generally applied to the entire population under study.
5. Does the project test a drug, device, biologic, some type of assay, or new medical software? If so, the project is likely human subjects research.
6. Was the project funded by a federal agency or by industry? While funding does not automatically classify a project as human subjects research, institutions must determine if the funding was awarded contingent on the project being conducted as a human subjects research study.
7. How are teams looking at the data? Typically, data are collected during human subjects research, and the analysis is done at the conclusion of the study. For quality improvement projects, data are typically reviewed and responded to continuously as a project moves forward. Plans for improvement change as learning occurs through each PDSA cycle. Evaluating change over time to determine if the change resulted in an improvement is a standard quality improvement data analysis method.
8. How are data analyzed? While not always the case, data analysis in a quality improvement project generally does not look for a difference in statistical significance between two treatment groups. More commonly, a quality improvement project analysis looks for differences in clinical significance to determine if the change being tested has resulted in improvement.
9. Is the project evidence-based? Quality improvement projects use evidence-based interventions with a reasonable expectation of improvement through participation. Quality improvement works to bring a clinic or procedure up to the current standard of care. On the other hand, the goal of human subjects research is to determine the efficacy of an intervention and possibly define a new standard of care or to determine the equivalency of treatments. Consequently, human subjects research may use experimental interventions that are not evidence-based.
10. What is the risk of participation? Typically, the only risk to participants in a quality improvement project is loss of privacy or confidentiality. The project team must implement measures to protect participant confidentiality and privacy. If the risk is more than minimal or more than is associated with usual care, including the unavoidable minimal risk in implementing any changes made in processes of care, the project is not a quality improvement project but human subjects research requiring informed consent.

Chapter 9, Ethical, Legal, and Regulatory Framework for Human Subjects Research, in the book *Optimizing the Nation's Investment in Academic Research: A New Regulatory Framework for the 21st Century*^10^ and the article “Quantitative Research Versus Quality Assurance, Quality Improvement, Total Quality Management, and Continuous Quality Improvement”^11^ provide additional information about human subjects research and quality improvement.

## CONCLUSION

Circling back to the two scenarios provided at the beginning of this article, the reader has likely determined that the immunization project is a quality improvement project and the study to determine if exercise reduces arthritis symptoms is human subjects research even though both projects seek to improve care. By applying the concepts and questions provided in this article, teams should be able to determine if their project is human subjects research, a quality improvement project, or both.

## Notes

This article meets the Accreditation Council for Graduate Medical Education and the American Board of Medical Specialties Maintenance of Certification competencies for Patient Care, Medical Knowledge, and Systems-Based Practice.
